# The effectiveness of syndromic surveillance for the early detection of waterborne outbreaks: a systematic review

**DOI:** 10.1186/s12879-021-06387-y

**Published:** 2021-07-20

**Authors:** Susanne Hyllestad, Ettore Amato, Karin Nygård, Line Vold, Preben Aavitsland

**Affiliations:** 1 Department of Infection Control and Preparedness, Norwegian Institute of Public Health, Oslo, Norway; 2 Faculty of Medicine, University of Oslo, Institute of Health and Society, Oslo, Norway

**Keywords:** Syndromic surveillance, Early detection, Gastrointestinal illness, Waterborne outbreaks

## Abstract

**Background:**

Waterborne outbreaks are still a risk in high-income countries, and their early detection is crucial to limit their societal consequences. Although syndromic surveillance is widely used for the purpose of detecting outbreaks days earlier than traditional surveillance systems, evidence of the effectiveness of such systems is lacking. Thus, our objective was to conduct a systematic review of the effectiveness of syndromic surveillance to detect waterborne outbreaks.

**Method:**

We searched the Cochrane Library, Medline/PubMed, EMBASE, Scopus, and Web of Science for relevant published articles using a combination of the keywords ‘drinking water’, ‘surveillance’, and ‘waterborne disease’ for the period of 1990 to 2018. The references lists of the identified articles for full-text record assessment were screened, and searches in Google Scholar using the same key words were conducted. We assessed the risk of bias in the included articles using the ROBINS-I tool and PRECEPT for the cumulative body of evidence.

**Results:**

From the 1959 articles identified, we reviewed 52 articles, of which 18 met the eligibility criteria. Twelve were descriptive/analytical studies, whereas six were simulation studies. There is no clear evidence for syndromic surveillance in terms of the ability to detect waterborne outbreaks (low sensitivity and high specificity). However, one simulation study implied that multiple sources of signals combined with spatial information may increase the timeliness in detecting a waterborne outbreak and reduce false alarms.

**Conclusion:**

This review demonstrates that there is no conclusive evidence on the effectiveness of syndromic surveillance for the detection of waterborne outbreaks, thus suggesting the need to focus on primary prevention measures to reduce the risk of waterborne outbreaks. Future studies should investigate methods for combining health and environmental data with an assessment of needed financial and human resources for implementing such surveillance systems. In addition, a more critical thematic narrative synthesis on the most promising sources of data, and an assessment of the basis for arguments that joint analysis of different data or dimensions of data (e.g. spatial and temporal) might perform better, should be carried out.

**Trial registration:**

PROSPERO: International prospective register of systematic reviews. 2019. CRD42019122332.

**Supplementary Information:**

The online version contains supplementary material available at 10.1186/s12879-021-06387-y.

## Background

Waterborne outbreaks have a particular high risk for public health, as exposure to drinking water that has been contaminated with pathogens could affect a large population in a relatively short period of time [1]. The early detection of infectious diseases is crucial to prevent related consequences, such as the loss of life, adverse health events, and societal burdens [2]. Moreover, experience has shown that relying only on the passive surveillance of laboratory-confirmed cases is not sufficient for the timely detection of waterborne outbreaks of non-endemic infections and may contribute to late detection and worse overall health impacts [3]. Syndromic surveillance (SyS), which aims to identify a threshold number of early symptomatic cases and facilitate the detection of an outbreak days earlier than conventional surveillance, has been implemented worldwide [4]. SyS is defined as the real-time (or near real-time) collection, analysis, interpretation and dissemination of health-related data [2], such as indicators of clinical signs and symptoms, as well as proxy measures such as over-the-counter pharmaceutical sales, hospital admission reports or infectious disease surveillance [5–8].

Although SyS has theoretical advantages in detecting waterborne outbreaks, the approach has been questioned in terms of the resources needed to evaluate the signals and distinguish them from “false alarms”, i.e. the specificity [9]. A number of factors is needed for evaluating public health surveillance system, including resources needed, usefulness, acceptability amongst other, in particular distinguishing an outbreak from a “false alarm” [10]. Some of the technical core assets to evaluate a surveillance system’s ability to detect a true outbreak is timeliness, sensitivity and specificity [11]. Useful surveillance systems for detecting a true outbreak is a balance between the timeliness, sensitivity and specificity, where the ideal situation is to have high values of sensitivity and specificity. However, in reality, this would require a less timely detection [9].

SyS systems for detecting waterborne outbreaks were reviewed in 2006 [12], with the recommendation that such surveillance should not be implemented at the expense of traditional surveillance. On the other hand, Berger et al. [12] also suggested that syndromic data sources, such as the over-the-counter sales of anti-diarrheal medications for detection of waterborne outbreaks, should be further evaluated [12]. In the aftermath of this review, several articles were published in the field of SyS for waterborne illness and early outbreak detection. However, these articles have not yet been reviewed for the purpose of assessing SyS effectiveness, indicating an updated knowledge gap in this field.

With this review, we aim to provide a knowledge update on the use and effectiveness of SyS approaches to detect waterborne outbreaks among populations connected to water supply systems earlier than traditional surveillance. We have specifically examined reported timeliness, sensitivity and specificity using implemented SyS approaches in contexts where health structures in place. An updated evidence for the effectiveness of the application of SyS will contribute to the evaluation and decision-making processes related to the implementation of this approach.

## Methods

### Literature search

We searched the Cochrane Library (http://www.thecochranelibrary.com/), Medline/PubMed (https://pubmed.ncbi.nlm.nih.gov/), EMBASE (https://www.embase.com/login), Scopus (http://www.Scopus.com), and Web of Science (https://apps.webofknowledge.com) for relevant published articles using a combination of the keywords ‘drinking water’, ‘surveillance’, and ‘waterborne disease’. A research librarian conducted the search between January to March of 2019, and the search strategy is described in Additional File 1. The publication period was set from 1990 to 2018, and only peer-reviewed publications in English, German, French, Spanish, and Scandinavian languages (Norwegian, Swedish, and Danish) were included in the search. The bibliographies of the eligible articles were screened to identify additional studies. We also searched Google Scholar for articles using the same key words to assess potential publications not identified in the bibliographic databases. The two latter searches were done to ensure an exhaustive search strategy until saturation was achieved [13].

### Selection criteria

We included studies on the early detection of waterborne outbreaks using signals from data sources other than diagnostic data. Descriptive and analytical studies (i.e. real-time outbreaks investigations or evaluation of data sources during previous outbreak situations) or simulation studies (i.e. testing systems using superimposed data or simulating cases for statistical/modelling purpose) on waterborne outbreaks were included in the review. Studies aiming at demonstrating a general association between gastrointestinal illness and drinking water exposure were excluded from the data synthesis, in addition to studies reporting health surveillance due to temporary emergency settings or as a response to natural disasters.

### Data extraction and analysis

The literature search output was uploaded in Rayyan [14], where the publications were screened for removing duplicates and processed for further screening. The Preferred Reporting Items for Systematic Reviews and Meta-Analyses (PRISMA) guidelines [15] were followed in the reporting of this review. Two reviewers independently screened the publications’ titles and abstracts against the inclusion criteria using the ‘blind-on’ function in Rayyan. Eligible studies for full-text review were further screened independently by two reviewers, and the following summary information was extracted and analysed from publications fulfilling the aim of the review: region/country, objective of study, study design, study period, outbreak cause, affected population, causative agents in the outbreak, and syndrome/data source for surveillance. A list of excluded studies with reasons for their non-inclusion is presented in Additional File 1.

The protocol of this systematic review was also approved by the National Institute of Health Research with the registration number PROSPERO 2019 CRD42019122332 and is available online (https://www.crd.york.ac.uk/prospero/display_record.php?RecordID=122332).

### Data synthesis

The information regarding effectiveness of the SyS in detecting waterborne outbreaks (i.e., timeliness, sensitivity, specificity) reported in the included articles was not suitable for pooling due to heterogeneity; therefore, a meta-analysis was not possible. A narrative summary of the findings of the timeliness of detection is presented as a summary in tabular form. Two researchers were involved in the data synthesis.

### Risk of bias in the individual studies and cumulative evidence

We used the Risk Of Bias In Non-randomized Studies of Interventions (ROBINS-I) assessment tool to assess the risk of bias in the individual studies [16]. The resulting body of evidence of the cumulative result was assessed by the Project on a Framework for Rating Evidence in Public Health (PRECEPT), which was developed by the European Centre for Disease Prevention and Control (ECDC) in 2012 [17, 18].

## Results

### Descriptive summary of study characteristics

**Fig. 1:**
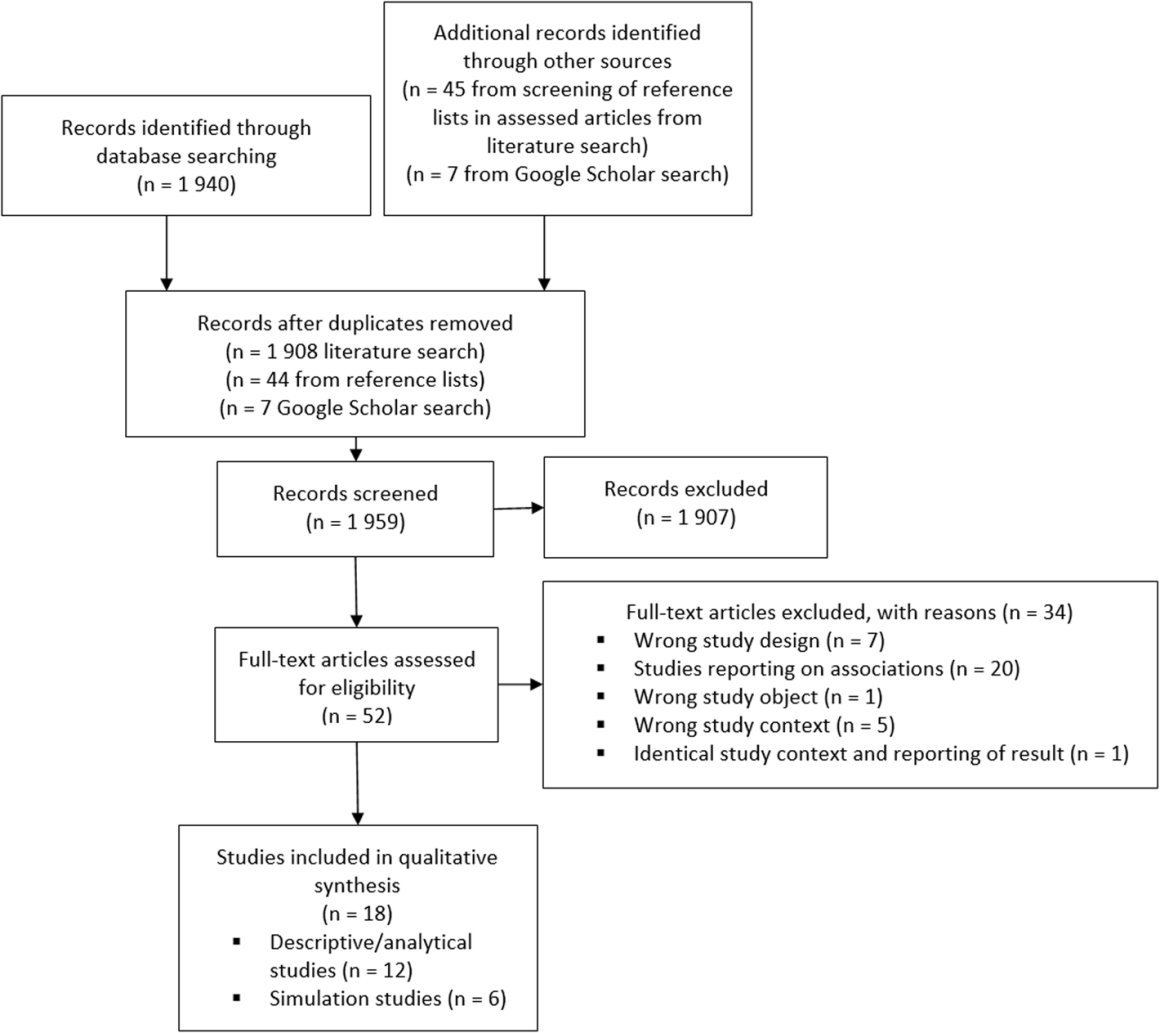
Flow of studies identified and screened in the review

From the 1959 articles identified in the literature search, screening of bibliographies (of the 27 articles found eligible for full-text screening in the literature search), and Google Scholar search, 18 articles were included in the review (Fig. 1). A summary of the study characteristics of the included studies is presented in Additional File 1. Of these included articles, 12 were descriptive or analytical studies assessing either historical outbreaks or data of cases of gastrointestinal illness and data signals for early detection of waterborne outbreaks [19–30], and six were simulation studies evaluating the system performance of different SyS systems [31–36]. The included studies originated from the USA (*n* = 7), France (*n* = 4), the United Kingdom (*n* = 3), Sweden (*n* = 2), Canada (*n* = 1) and, with one study assessing data from several European countries suggesting a common surveillance approach [24], covering an overall study period of 1997 to 2013, with multiple agents causing waterborne outbreaks or illness. Twelve of the included studies were published in the period 2010 to 2018, five that was published in 2004–2006 and one in 1998 (Additional File 1).

Among the excluded articles, the majority were data signal studies, including investigations of water quality data or disturbances in the distribution systems, in combination with other signals from the health sector [37–46]. One study used web queries to estimate the burden of disease due to gastrointestinal illness related to pipe breaks [7], while another study assessed the relationship between precipitation and waterborne diseases [47]. Common in these studies was the fact that, despite that they demonstrated promising correlations, they did not report on the experienced effectiveness or value of using the same signals in their surveillance system explicitly. The other excluded studies addressed SyS but in the context of describing or reviewing such systems in a general manner [48, 49], or gastrointestinal cases without assessing for the detection of waterborne outbreaks in particular [50, 51] (Additional File 1).

### Data synthesis

The data extracted from the included articles is synthesized in Table 1. When reported, the sensitivity of the SyS in the retrospective studies was below 50%. In the simulation studies, the sensitivity was reported above 70% when using different aberration adjustments.

**Table 1:** Synthesis of data from the included articles (*n* = 18)

Data signal	Reference	Timeliness	Sensitivity/Specificity	Pros	Cons
**Single data signal SyS system**					
Over-the-counter (OTC) sales of pharmacy sales	Edge et al., 2004 [30]^a^	NI	NI	In situations where infected individuals have symptoms prompting self-medication, OTC sales trend would provide a more sensitive, timely and geographically specific detection tool than monitoring emergency room visits and laboratory-based surveillance.	Adaptations to the algorithm will have to be developed to adjust for a number of factors contributing to the general noisiness of these data such as seasonal effects, promotional sales and type of population served. The success of such system will rely on automatic collection, analysis and dissemination of results.
	Kirian et al., 2011 [25]^a^	NI	Sensitivity: 4-14%, specificity: 97-100%.	It may capture symptoms in the population before a person with gastrointestinal illness seeks health care.	It does not necessarily indicate the buyer’s location, their demographic status, or the reason for the purchase. Those who purchase OTC medications for their illness may not be representative of the sick population as a whole. Hoarding behaviour will also affect the outcome.
Reimbursement of prescription drugs	Mouly et al., 2016 [20]^a^	NI	Sensitivity: 6 and 21% for two examined outbreaks.	Prescription drug data can be considered for the development of a detection system of waterborne outbreaks given its ability to describe an epidemic signal. It could support authorities in slow developing outbreaks.	The algorithm cannot be used directly in other countries because of their different health systems, types and sources of data, and medical practices. The accuracy depends on the medical consultation rate in the impacted population. The accuracy of using health insurance data to describe waterborne outbreaks depends on the medical consultation rate in the impacted population, however, as this is never the case, data analysis underestimates the total number of acute gastrointestinal cases.
Calls to health advice line (‘telehealth’)	Bjelkmar et al., 2017 [21]^a^	~ 2,5 months	NI	Comparing call patterns between water distribution areas that were based on groups of postal codes gives timely indication of the underlying cause and therefore substantially increases the chances of effective countermeasures	Tradeoff between sensitivity and specificity in signal detection. Need for a protocol for signal evaluation and validation, especially for regions where the population size is small.
**Multiple data signal SyS systems**					
Emergency care data; medical dispatch, ambulance medical service, emergency department chief complaints	Balter at al., 2005 [27]^a^	NI	NI	Emergency department syndromic surveillance might prove useful for detecting a problem and quantifying its magnitude.	This system cannot determine the true etiology. If insufficient information exists to initiate an investigation, the decision is often made to observe whether the signal continues the next day, thereby losing syndromic surveillance’s theoretical advantage of timeliness.
	Ziemann et al., 2014 [24]^a^	NI	NI	This system could detect changes in local trends and clusters of statistical alarms.	It is not likely to detect local gastrointestinal outbreaks with few, mild, or dispersed cases. The probability of detecting an outbreak increases with the outbreak size. The results cannot be generalized to region-level data or very sparse time series.
Over-the-counter (OTC), web queries, calls to health advice line	Andersson et al., 2014 [19]^a^	NI	Calls to health advice line: sensitivity: 40-50%, specificity: 99%, web queries and OTC: no signal.	SyS can serve as an early warning for waterborne outbreaks, especially with telephone triage data with sufficient temporal and spatial resolution. It may be suited to detecting widespread rises in syndromes and, rarely, small-scale outbreaks.	The alarm does not contain information on the cases’ medical status to validate the cause of the alarm. Moderate and low outbreaks (< 1000 cases) are unlikely to be detected. Limitations to the reported results are linked to one of the four outbreaks were not waterborne.
Telehealth, in-hours and out-of-hours GP, ED visits	Smith et al., 2010 [22]^a^	Peak of calls coincides with outbreak (95% CI) in one area	NI	Multiple syndromic data streams are an advantage.	Telehealth may, in general, be driven by media bias.
Chief complaints of patients reporting to emergency departments, over-the-counter and prescription pharmacy sales, and worker absenteeism	Heffernan et al., 2004 [28]^a^	NI	NI	Syndromic surveillance systems have proved useful for detecting substantial citywide increases in common viral illnesses (e.g. influenza, norovirus and rotavirus).	The studied systems have not detected more contained outbreaks earlier than traditional surveillance.
Combined health, spatial and environmental data	Proctor et al., 1998 [29]^a^	Timeliness of learning about the peak was 15 days earlier in in monitoring treatment plant effluent turbidity compared to ER’s visits and clinical laboratory.	NI	It is noted the value of alternate data sources as early warning systems which can complement laboratory diagnosis.	There are weaknesses for all proposed surrogate waterborne surveillance systems. For example, turbidity did not give information on disease causing-organisms; and treated water meeting quality standard could still contain sufficient level of pathogens.
	Rambaud et al., 2016 [26]^a^	NI	NI	Combining two complementary methods protects against false positives, e.g. confusion of cases stemming from exposure from other types of food or swimming, for example.	Pilot-study and not tested on a larger scale.
	Coly et al., 2017 [23]^a^	NI	Detected outbreaks < 100 cases.	Increases sensitivity and timely detection of waterborne outbreaks.	These systems are expensive in terms of resources and shared expertise in incorporating local knowledge regarding both environmental and health data.
**Simulations**					
Method evaluations via simulations of multiple signal SyS systems	Cooper et al., 2006 [36]^b^	Unlikely to detect local outbreak	NI	It may capture symptoms in the population before seeking health care.	The alarm does not contain information regarding the cases’ medical status to validate the cause of the alarm. Moderate and low outbreaks (< 1000 cases) are unlikely to be detected. The detection ability varies seasonally. Telehealth may, in general, be driven by media bias.
	Burkom et al., 2011 [31]^b^	NI	Sensitivity: 80%, specificity: 99%	Use of multiple syndromic data streams is an advantage. The number of false alarms is greatly reduced.	Simulation results must generally be improved with real epidemiological data.
	Xing et al., 2011 [35]^b^	NI	Of the simulated models, the regression method had higher sensitivity (range 6–14% improvement of sensitivity in the surveillance system).	Demonstrates possible improvement in the surveillance system to increase sensitivity.	Simulations based on small number of data points.
	Zhou et al., 2015 [34]^b^	3.3 to 6.1 days	When reported, the sensitivity ranged from 24 to 77%, and the PPV was 90.5%.	Sensitivity and timeliness increase with stratification.	Study population perhaps not representative.
	Colón-Gonzales et al., 2018 [33]^b^	Unlikely to detect outbreaks < 1000 cases	NI	Framework applicable for other SyS systems.	The detection ability varies seasonally.
	Mouly et al., 2018 [32]^b^	NI	Sensitivity: 73%, PPV: 90.5%	Space-time increases the likelihood of detecting outbreaks.	The probability of detecting outbreaks increases with the outbreak size.

*NI* not identified, *PPV* positive predictive value

^a^descriptive and analytical study based on historical data

^b^simulation study using different aberration for system performance

Some of the included studies addressed the same surveillance system but with different study purposes. In France, a national surveillance system based on administrative health data from the French National Health Insurance on the reimbursement of prescriptive drugs has been functioning since the late 1990s [51]. The system contains information on the medications for gastrointestinal illness, which are reimbursable, prescribed by a general practitioners (GPs) and dispensed in a pharmacies covering approximately 98% of the French population [23].. All the included articles originating from French study data were related to this health administrative database.

In the UK, the SyS at Public Health England (PHE) is based on four National Health Service (NHS) healthcare settings: telehealth, in- and out-of-hours, unscheduled care general practitioner consultations, and emergency department (ED) attendances [33]. This system has been examined, together with the of the Health Protection Agency (HPA) and QSurveillance, a national surveillance system set up by the University of Nottingham, and the Egton Medical Information System, which consists of a network of GPs [22].

In the US, several surveillance systems exist [43, 48, 49], and, in this review, we included publications addressing the Electronic Surveillance System for the Early Notification of Community-Based Epidemics (ESSENCE) [31]. Additionally, two studies assessed the US Centers for Disease Control and Prevention BioSense surveillance system using emergency department chief complaint data [35] and daily syndrome counts from the outpatients of the U.S. Department of Veteran Affairs’ Veteran Health Administration [34]. Moreover, both of the two included studies from Sweden addressed data signals from Swedish Health Care Direct 1177 (Vårdguiden 1177), along with signals such as web queries and over-the-counter pharmacy sales in one of the study [19, 21].

### Single data signal SyS system

Five of the included studies addressed a single preclinical data signal for outbreak detection and gastrointestinal illness. In 2004, Edge et al. [30] evaluated the potential of a syndromic surveillance system by comparing retrospective pharmacy OTC sales of anti-nauseants and anti-diarrheals to emergency room visits and case numbers from two Canadian outbreaks. The authors concluded that spatial and temporal trend analyses of daily OTC sales would provide supplemental community health information for public health officials that is timelier than currently available laboratory-based surveillance systems. Kirian et al. [25] evaluated the ability of drug sales in predicting endemic and epidemic gastrointestinal disease in the San Francisco area and found no significant correlations between drug sales and illness case counts, outbreak counts, or the number of outbreak-associated cases and reported a low sensitivity (4–14%) and high specificity (97–100%) in the study [25].

Mouly et al. [20] conducted a comparative study of two waterborne outbreaks from cohort studies with health administrative databases. Almost three-quarters of the simulated outbreak were detected, estimated a sensitivity of 73%, and more than 9 out of 10 detected signals corresponded to a waterborne outbreak (PPV 90.5%). The authors reported in addition that probability to detect an outbreak increase with outbreak size.

Bjelkmar et al. [21] extended on such a system for nurse health calls proposed by Andersson et al. [19]. The authors compared phone call patterns to the Swedish Health Care Direct 1177 during the outbreak in Skellefteå in different water distribution areas. They suggested that – under the scenario that if the outbreak had been detected earlier and assuming that all cases from 1st February forward had remained healthy – the systematic monitoring of phone calls made to health services could have limited the outbreak from 18,500 cases to approximately 2300 cases by detecting the outbreak approximately 2.5 months earlier than actually detected [21].

### Multiple data signal SyS systems

The earliest published study included in this review is Proctor et al. [29], whom assessed eight different data sources available during the time of the *Cryptosporidium* outbreak in Milwaukee in 1993, and described the relative strengths and weaknesses of these surveillance methods. During the investigation, surveillance systems which could be easily linked with laboratory data, were flexible in adding new variables, and which demonstrated low baseline variability were most useful. However, although there was a remarkable temporal correspondence of surveillance peaks, the most timely data involved use of systems in which personnel with existing close ties to public health programs perceived the importance of providing information despite workload constraints associated with an outbreak [29].

Heffernan et al. [28] and Balter et al. [27] both describe and evaluate the experienced usefulness of using syndromic surveillance for the detection of waterborne outbreaks in New York City. Balter et al. report on syndromic surveillance using multiple health data sources, while Heffernan et al. report on the same system utilizing data such as chief complaints of patients reporting to emergency departments, over-the-counter and prescription pharmacy sales, and worker absenteeism. In both publications, the authors report that it have not detected waterborne outbreaks earlier that traditional surveillance and should be viewed as supplement to well-maintained traditional surveillance systems.

For establishing a national SyS system, Andersson et al. [19] evaluated the efficiency of alternate data sources for the early detection of nine investigated outbreaks in Sweden, of which three were large waterborne outbreaks, including telephone triage, web-queries, and over-the-counter (OTC) pharmacy sales. The authors suggested, after assessing the three waterborne outbreaks and an additional foodborne outbreak, that SyS can serve as an early warning of outbreaks, especially with telephone triage data with sufficient temporal and spatial resolution (40–50% sensitivity and 99% specificity); however, data was lacking for outbreaks of moderate size (300–1000 cases) [19].

Smith et al. [22] evaluated the value of SyS in monitoring small waterborne outbreaks using data from a SyS system featuring a direct telephone helpline and QSurveillance national SyS using clinical diagnosis data extracted from the GP clinical information system [22]. The authors reported that, for the first time, such a SyS system was helping to monitor a small-scale waterborne outbreak; however, the peaks of calls to the helpline observed may have been influenced by the media as a boil water advisory was issued during the outbreak [22].

Using routine emergency data based on an inventory of sub-national emergency data available in 12 European countries, Ziemann et al. [24] proposed a framework of definitions for specific symptoms and a SyS system design applying cumulative sum and spatial-temporal cluster analyses for the detection of local gastrointestinal outbreaks in four countries. Based on the suggested system, the authors identified two gastrointestinal outbreaks in two countries, and 1 out of the 147 confirmed outbreaks in the studied countries was detected [24].

### Combined SyS systems with environmental data

Two articles included in this review combined water quality data and information on supply zones in the SyS in France. A pilot study was conducted by Rambaud et al. [26] to assess the utility of using a health insurance database for the automated detection of waterborne outbreaks of acute gastroenteritis [26]. Overall, 193 clusters were identified, with 10% of the municipalities involved in at least one cluster and less than 2% in several [26]. To improve the detection of waterborne outbreaks, Coly et al. [23] developed an integrated approach to detect any study clusters of acute gastrointestinal infection in geographical areas with a homogeneous exposure to drinking water. They used data from the French SyS system, geographical and population data, and environmental data based and the application of a space-time detection method identified 11 potential waterborne disease outbreaks. The outbreaks identified were not investigated, but the risk factors of exposure were examined [23].

### Method evaluations via simulations

Three of the included articles concerned simulations of SyS systems in the US. Burkom et al. [31] studied an integrated approach for the fusion of water quality data (e.g., faecal indicator bacteria, chlorine, pH, conductivity, and turbidity) with health monitoring data (ESSENCE) using probabilistic Bayesian networks. The simulations indicated a sensitivity of 80% and specificity of 99% for the symptoms “nausea/vomit” [31], however, further component simulations and the multidisciplinary development of realistic data scenarios would be needed [31]. Xing et al. [35] compared timeliness of the SyS system using five regression models, and found that the sensitivity for ‘nausea and vomiting’ was calculated to approximately 55% [35]. The simulations in the study of Xing et al. [35] had a number of limitations, including a low number of data points. Zhou et al. [34] examined the performance of the U.S. Centers for Disease Control and Prevention’s BioSense SyS system by injecting multi-day signals stochastically drawn from lognormal distributions into time series of aggregated daily visit counts for the outpatients at the Department of Veterans Affairs’ Veterans Health Administration (VHA) [34]. The authors reported that, with a daily background alert rate of 1 and 2%, the sensitivities and timeliness in the SyS ranged from 24 to 77% and 3.3 to 6.1 days, respectively [34].

In the UK, two published studies presented measures to improve the method performance of national SyS systems. In Cooper et al. [36], included in this review, calls made to the health helpline (NHS Direct) were assessed based on whether the number of calls about diarrhoea exceeded a statistical threshold [36]. The authors predicted a 4% chance of detection when assumed that one-twentieth of cryptosporidiosis cases telephoned the helpline, which rose to a 72% chance when assumed nine-tenths of cases telephoned. They concluded that NHS Direct was currently unlikely to detect an event similar to the cryptosporidiosis outbreak used in the study and may be most suited to detecting more widespread increases in symptoms [36].

Colón-Gonzales et al. [33] investigated how the characteristics of different outbreaks affected outbreak detection and the utility of SyS in detecting outbreaks using modelling and probability/statistics for two possible scenarios, including a localized outbreak of cryptosporidiosis. The authors reported that small gastrointestinal outbreaks (e.g., cryptosporidiosis) were unlikely to be detected unless the number of cases was over 1000, with the detection of waterborne outbreaks varying by season [33]. Multiple data streams (e.g., emergency attendance) are an advantage of influenza detection but not for outbreaks of cryptosporidiosis. However, the proposed framework of Colón-Gonzales et al. (2018) could, according to the authors, be applicable for the evaluation of any SyS system [33].

Mouly et al. 2018 [32] evaluated the performance of an algorithm using the French SyS system for waterborne outbreak detection through a simulation-based study using multivariate regression to identify the factors associated with outbreak detection. Almost three-quarters of the simulated outbreak were detected (sensitivity of 73%), and more than nine out of the 10 detected signals corresponded to a waterborne outbreak (positive predictive value of 90.5%). The probability of detecting an outbreak was found to increase with the outbreak size [32].

### Risk of bias and cumulative body of evidence

The risk of bias of the included studies was overall assessed to be moderate to serious (Additional File 1). Due to the heterogeneity of the articles included, the cumulative body of evidence was partly assessed using the PRECEPT framework. The evidence was graded as high due to the low risk of publication bias.

## Discussion

In this systematic review, we identified 12 articles assessing the detection of waterborne outbreaks using different syndromic surveillance systems and six articles simulating a detection using a variation of statistical methods for the system performance improvements. The articles originated from four countries and represented five systems.

### Effectiveness of SyS systems in detecting waterborne outbreaks

The results reported in the included articles are generally modest (sensitivity below 50%) in their ability to detect waterborne outbreaks regardless of data signals. However, the simulation studies included in this review imply that multiple sources of signals combined with spatial information may increase the sensitivity in the SyS system of detecting waterborne outbreaks and reduce false alarms. The effectiveness of a SyS is a balance between sensitivity, specificity and predictive value, and timeliness, implying that high sensitivity may lead to a less timely detection [9]. Because surveillance systems vary widely in terms of methodology, scope, and objectives, the characteristics that are important to one system may be less important to another. Efforts to improve certain attributes, such as the ability of a system to detect a health event (sensitivity), may detract from other attributes, such as simplicity or timeliness [10].

The use of over-the-counter pharmacy sales have been reported as not useful to detect waterborne outbreaks [25], while others have reported its usefulness [8]. Drug sales data analysis for the outbreak detection of infectious diseases was reviewed in 2014 by Pivette et al. [8], with the conclusion that over-the-counter sales appear to be a useful tool in detection trends gastrointestinal disease [8]; however, the review may have been prone to publication bias. Only a few studies have shown promising correlations between SyS and signals, such as those originating from contact for health consultations in the health care system [21, 52]. Such conflicting reporting of results should not be surprising, since the SyS systems included in the review are context-specific and not directly comparable. Although this review provides an updated overview of published articles assessing the effectiveness of SyS in detecting waterborne outbreaks, the synthesis of the articles was challenging, since they varied greatly in terms of administrative and geographical context, the data signals and algorithms used, and how the results were reported. The effectiveness of SyS system, in general, also largely relies on the methods used to detect aberrations.

The timeliness of surveillance approaches for outbreak detection is the amount of time from exposure to the disease agent to the initiation of a public health intervention [10]. Berger et al. scored environmental data in terms of timeliness from a range of typical data used for SyS [12]. However, when observing a change in for instance environmental data that may affect public health, disease in the population is less likely to have been developed. In drinking water supply systems, there is an obligation of the water supplier to prompt action to mitigate a breach exceedance in the monitoring of microbiological parameters. Often, the mitigating action is the issuance of a boil water advisory to protect the population from a potentially evolving outbreak [53]. The risk of developing a waterborne outbreak is higher when a contamination event goes undetected during day-to-day-operations and routine monitoring, which is a common factor in several waterborne outbreaks [54, 55]. In some of the identified articles included in this review, the benefit of combining the surveillance system to geographical supply zones to increase the likelihood of detecting a waterborne outbreak was highlighted. On the other hand, deploying such systems may be challenging, since it will most likely involve two different fields of expertise (health and technical), and the processing of data to inform health decisions must still be accounted for, since local outbreaks are usually short-lived [56].

The association between gastrointestinal illness cases and water quality data, such as turbidity, has been reviewed by de Roos et al. [57] in an attempt to discern the presence of waterborne gastrointestinal illness. However, the utility of turbidity as a proxy for microbiological contamination may be context-specific [57]. Several of the excluded articles (Additional File 1) examined the potential of strengthening surveillance by including water quality data. In particular, these included, turbidity, disturbances in the distribution network, and calls to an alarm centre, among others. However, none of the excluded studies reported on analysis linked to real or simulated outbreaks. One of the most reported causes of waterborne outbreaks is heavy rainfall, which represents a future increasing risk [58] and implies a greater call for a risk-based approach to surveillance for water supply systems [56]. In general, since there will always be a risk of water contamination going undetected, prioritizing long-term preventive measures and risk-based surveillance should not be underestimated despite promising reporting on SyS systems.

### Strengths and limitations

There are several limitations related to our review. First, the rather wide scope of the review resulted in a variety of articles that may have been of interest to the study topic but were excluded due to a lack of eligibility. Still, the included articles were also different from each other in many ways and did not allow for an accurate comparison of the reported results and assessments of the risk of bias. We also only found articles from five countries representing five surveillance systems, which limited the possibility of generalizing the results in terms of effectiveness to detect waterborne outbreaks. However, there might be scope for a more critical thematic narrative synthesis on the most promising sources of data, and an assessment of the basis for arguments that joint analysis of different data or dimensions of data (e.g. spatial and temporal) might perform better.

In general, observational and retrospective studies are more prone to bias than randomized controlled studies (RCTs) due to a lack of randomization and blinding, hence jeopardizing their external and internal validity, which also affected the general outcome of the review. The ROBINS-I tool used in this review could only be partly used for the assessment of the risk of bias. We assessed risk of bias on a more-or-less hypothetically manner of the studies since developing the mimic RCT according to Sterne et al. [16], was challenging. Studies examining the detection of waterborne outbreaks based on real investigated outbreaks generally were assessed as having a lower bias due to confounding than those only using data on water quality deviations as a risk factor for waterborne illness. Bias in the selection of participants was, in general, a problem in the observational studies. In this review, all the studies using register data were assessed as having a lower risk of bias have been rated as having a moderate or serious risk of bias. Moreover, bias in the classification of interventions was different among the studies examining outbreaks as serious due to the risk of differential misclassification (recall bias), while studies using register data with confirmed aetiology had a decreased risk of classification bias; however, there was a lack of evidence on illness attributed to drinking water. Bias in the domains of deviation from the intended interventions, missing data, and the measurement of outcomes were regarded as not applicable to this review. Bias in the selection of the reported results was assessed as serious in cases in which only one data signal was studied.

A strength of this review is its comprehensive search of published peer-reviewed articles using multiple databases, the screening of bibliographies, and a Google Scholar search of the topic of SyS systems’ effectiveness in detecting waterborne outbreaks. The screening of bibliographies is a ‘snow-balling’ technique that entails a targeted assessment of the topic. The fact that there only a minor contribution of publications stemming from the Google Scholar search, may be interpreted that we had identified the relevant publications mainly through bibliographical search.

## Conclusion

Waterborne outbreaks still represent a risk in developed countries, and their early detection is crucial for the prevention of societal consequences. SyS systems with different features are widely used for the detection of waterborne outbreaks; however, in this review, we did not find evidence for syndromic surveillance in terms of their ability to effectively detect waterborne outbreaks (low sensitivity and high specificity), especially small and localized outbreaks. There are, on the other hand, promising development towards surveillance systems combining health, geographic, and water quality data; however, such systems must be evaluated in a cost-benefit context. This review demonstrates that there is no conclusive evidence regarding the effectiveness of SyS for the detection of waterborne outbreaks, which also emphasizes the need to focus on primary prevention measures to reduce the risk of waterborne outbreaks and risk-based surveillance. Future studies should include methods for combining health and environmental data with an assessment of the resources required for operating such a system. In addition, a more critical thematic narrative synthesis on the most promising sources of data, and an assessment of the basis for arguments that joint analysis of different data or dimensions of data (e.g. spatial and temporal) might perform better, should be carried out.

## Supplementary Information

**Additional file 1.** Literature search strategy and results.

## Data Availability

Additional file 1 with literature search and excluded articles is made available in Additional file 1.
