# Correction to: The effectiveness of syndromic surveillance for the early detection of waterborne outbreaks: a systematic review

**DOI:** 10.1186/s12879-021-06843-9

**Published:** 2022-01-05

**Authors:** Susanne Hyllestad, Ettore Amato, Karin Nygård, Line Vold, Preben Aavitsland

**Affiliations:** 1 Department of Infection Control and Preparedness, Norwegian Institute of Public Health, Oslo, Norway; 2 Faculty of Medicine, University of Oslo, Institute of Health and Society, Oslo, Norway

## Correction to: BMC Infect Dis (2021) 21:696 https://doi.org/10.1186/s12879-021-06387-y

Following the publication of the original article [1], some errors were identified in the text and in Table 1:

The sentences currently read:

- the systematic monitoring of phone calls made to health services could have limited the outbreak from 18,500 cases to approximately 2300 cases by detecting the outbreak approximately six days earlier than actually detected

The sentence should read:

- the systematic monitoring of phone calls made to health services could have limited the outbreak from 18,500 cases to approximately 2300 cases by detecting the outbreak approximately 2.5 months earlier than actually detected.

Furthermore, the correct Table 1 is given in this Correction article.

**Table 1:** Synthesis of data from the included articles (n = 18)

Data signal	Reference	Timeliness	Sensitivity/Specificity	Pros	Cons
*Single data signal SyS system*					
Over-the-counter (OTC) sales of pharmacy sales	Edge et al., 2004 [30]*	NI	NI	In situations where infected individuals have symptoms prompting self-medication, OTC sales trend would provide a more sensitive, timely and geographically specific detection tool than monitoring emergency room visits and laboratory-based surveillance	Adaptations to the algorithm will have to be developed to adjust for a number of factors contributing to the general noisiness of these data such as seasonal effects, promotional sales and type of population served. The success of such system will rely on automatic collection, analysis and dissemination of results
	Kirian et al., 2011 [25]*	NI	Sensitivity: 4–14%, specificity: 97–100%	It may capture symptoms in the population before a person with gastrointestinal illness seeks health care	It does not necessarily indicate the buyer’s location, their demographic status, or the reason for the purchase. Those who purchase OTC medications for their illness may not be representative of the sick population as a whole. Hoarding behaviour will also affect the outcome
Reimbursement of prescription drugs	Mouly et al., 2016 [20]*	NI	Sensitivity: 6% and 21% for two examined outbreaks	Prescription drug data can be considered for the development of a detection system of waterborne outbreaks given its ability to describe an epidemic signal. It could support authorities in slow developing outbreaks	The algorithm cannot be used directly in other countries because of their different health systems, types and sources of data, and medical practices. The accuracy depends on the medical consultation rate in the impacted population. The accuracy of using health insurance data to describe waterborne outbreaks depends on the medical consultation rate in the impacted population, however, as this is never the case, data analysis underestimates the total number of acute gastrointestinal cases
Calls to health advice line (‘telehealth’)	Bjelkmar et al., 2017 [21]*	~ 2.5 months	NI	Comparing call patterns between water distribution areas that were based on groups of postal codes gives timely indication of the underlying cause and therefore substantially increases the chances of effective countermeasures	Tradeoff between sensitivity and specificity in signal detection. Need for a protocol for signal evaluation and validation, especially for regions where the population size is small
*Multiple data signal SyS systems*					
Emergency care data; medical dispatch, ambulance medical service, emergency department chief complaints	Balter et al., 2005 [27]*	NI	NI	Emergency department syndromic surveillance might prove useful for detecting a problem and quantifying its magnitude	This system cannot determine the true etiology. If insufficient information exists to initiate an investigation, the decision is often made to observe whether the signal continues the next day, thereby losing syndromic surveillance's theoretical advantage of timeliness
	Ziemann et al., 2014 [24]*	NI	NI	This system could detect changes in local trends and clusters of statistical alarms	It is not likely to detect local gastrointestinal outbreaks with few, mild, or dispersed cases. The probability of detecting an outbreak increases with the outbreak size. The results cannot be generalized to region-level data or very sparse time series
Over-the-counter (OTC), web queries, calls to health advice line	Andersson et al., 2014 [19]*	NI	Calls to health advice line: sensitivity: 40–50%, specificity: 99%, web queries and OTC: no signal	SyS can serve as an early warning for waterborne outbreaks, especially with telephone triage data with sufficient temporal and spatial resolution. It may be suited to detecting widespread rises in syndromes and, rarely, small-scale outbreaks	The alarm does not contain information on the cases’ medical status to validate the cause of the alarm. Moderate and low outbreaks (< 1000 cases) are unlikely to be detected. Limitations to the reported results are linked to one of the four outbreaks were not waterborne
Telehealth, in-hours and out-of-hours GP, ED visits	Smith et al., 2010 [22]*	Peak of calls coincides with outbreak (95% CI) in one area	NI	Multiple syndromic data streams are an advantage	Telehealth may, in general, be driven by media bias
Chief complaints of patients reporting to emergency departments, over-the-counter and prescription pharmacy sales, and worker absenteeism	Heffernan et al., 2004 [28]*	NI	NI	Syndromic surveillance systems have proved useful for detecting substantial citywide increases in common viral illnesses (e.g. influenza, norovirus and rotavirus)	The studied systems have not detected more contained outbreaks earlier than traditional surveillance
Combined health, spatial and environmental data	Proctor et al., 1998 [29]*	Timeliness of learning about the peak was 15 days earlier in in monitoring treatment plant effluent turbidity compared to ER’s visits and clinical laboratory	NI	It is noted the value of alternate data sources as early warning systems which can complement laboratory diagnosis	There are weaknesses for all proposed surrogate waterborne surveillance systems. For example, turbidity did not give information on disease causing-organisms; and treated water meeting quality standard could still contain sufficient level of pathogens
	Rambaud et al., 2016 [26]*	NI	NI	Combining two complementary methods protects against false positives, e.g. confusion of cases stemming from exposure from other types of food or swimming, for example	Pilot-study and not tested on a larger scale
	Coly et al., 2017 [23]*	NI	Detected outbreaks < 100 cases	Increases sensitivity and timely detection of waterborne outbreaks	These systems are expensive in terms of resources and shared expertise in incorporating local knowledge regarding both environmental and health data
*Simulations*					
Method evaluations via simulations of multiple signal SyS systems	Cooper et al., 2006 [36]**	Unlikely to detect local outbreak	NI	It may capture symptoms in the population before seeking health care	The alarm does not contain information regarding the cases’ medical status to validate the cause of the alarm. Moderate and low outbreaks (< 1000 cases) are unlikely to be detected. The detection ability varies seasonally. Telehealth may, in general, be driven by media bias
	Burkom et al., 2011 [31]**	NI	Sensitivity: 80%, specificity: 99%	Use of multiple syndromic data streams is an advantage. The number of false alarms is greatly reduced	Simulation results must generally be improved with real epidemiological data
	Xing et al., 2011 [35]**	NI	Of the simulated models, the regression method had higher sensitivity (range 6–14% improvement of sensitivity in the surveillance system)	Demonstrates possible improvement in the surveillance system to increase sensitivity	Simulations based on small number of data points
	Zhou et al., 2015 [34]**	3.3 to 6.1 days	When reported, the sensitivity ranged from 24 to 77%, and the PPV was 90.5%	Sensitivity and timeliness increase with stratification	Study population perhaps not representative
	Colón-Gonzales et al., 2018 [33]**	Unlikely to detect outbreaks < 1000 cases	NI	Framework applicable for other SyS systems	The detection ability varies seasonally
	Mouly et al., 2018 [32]**	NI	Sensitivity: 73%, PPV: 90.5%	Space–time increases the likelihood of detecting outbreaks	The probability of detecting outbreaks increases with the outbreak size

*Descriptive and analytical study based on historical data, **simulation study using different aberration for system performance

Note: NI = not identified, PPV = positive predictive value

The original article [1] has been corrected.
