# Supplementary material for: The effectiveness of syndromic surveillance for the early detection of waterborne outbreaks: a systematic review
Source: BMC Infect Dis. 2021 Jul 20;21:696. doi: 10.1186/s12879-021-06387-y (PMC8290622; doi:10.1186/s12879-021-06387-y)
Supplement: Supplementary file 1 — Additional file 1. Literature search strategy and results. [file 12879_2021_6387_MOESM1_ESM.docx]

The effectiveness of syndromic surveillance for the early detection of waterborne outbreaks: a systematic review

PROSPERO ID: CRD42019122332

Susanne Hyllestad*^1,2^, Ettore Amato^1^, Karin Nygård^1^, Line Vold^1^, Preben Aavitsland^1^

^1^Norwegian Institute of Public Health, Department of Infection Control and Preparedness, Oslo, Norway

^2^University of Oslo, Faculty of Medicine, Institute of Health and Society, Oslo, Norway

*Corresponding author: Norwegian Institute of Public Health, [Susanne.hyllestad@fhi.no](mailto:Susanne.hyllestad@fhi.no)

# Additional file 1

## Literature search strategy

Database: MEDLINE

Date: 27.03.2019

Number of identified articles: 687

| **#** | **Searches** | **Results** |
| --- | --- | --- |
| 1 | Drinking water/ or Water supply/ or Water*.tw,kf. | 763557 |
| 2 | Sentinel surveillance/ or POPULATION SURVEILLANCE/ or (syndromic surveillance* or Sentinel surveillance* or (early adj2 outbreak*) or "early warning*" or "risk based surveillance*").tw,kf. | 68165 |
| 3 | Waterborne Diseases/ or (outbreak* or waterborne or "water borne" or infection* or infectious* or "water related disease*").tw,kf. | 1481492 |
| 4 | 1 and 2 and 3 | 687 |

Database: Embase

Date: 27.03.2019

Number of identified articles: 999

| **#** | **Searches** | **Results** |
| --- | --- | --- |
| 1 | drinking water/ or water supply/ or Water*.tw,kw. | 886634 |
| 2 | sentinel surveillance/ or health survey/ or (syndromic surveillance* or Sentinel surveillance* or (early adj2 outbreak*) or "early warning*" or "risk based surveillance*").tw,kw. | 192928 |
| 3 | water borne disease/ or (outbreak* or waterborne or "water borne" or infection* or infectious* or "water related disease*").tw,kw. | 1821962 |
| 4 | 1 and 2 and 3 | 999 |

Database: Scopus

Date: 10.04.2019

Number of identified articles: 111

| 5 | #4 AND NOT  INDEX ( medline ) | 111 |
| --- | --- | --- |
| 4 | #1 AND #2 AND # 3 | 287 |
| 3 | ( KEY ( "Waterborne Diseases"  OR  "Waterborne Disease"  OR  "Water borne Diseases"  OR  "Water borne Disease" )  OR  TITLE-ABS ( outbreak*  OR  outbreaks  OR  waterborne  OR  "water borne"  OR  infection  OR  infections  OR  infectious*  OR  "water related disease"  OR  "water related diseases" ) ) | 1,807,212 |
| 2 | ( KEY ( «sentinel  AND surveillance»  OR  «population  AND surveillance» )  OR  TITLE-ABS ( "syndromic surveillance"  OR  "syndromic surveillances"  OR  "Sentinel surveillance"  OR  "Sentinel surveillances"  OR  ( early  W/1  outbreak )  OR  ( early  W/1  outbreaks )  OR  "early warning"  OR  "early warnings"  OR  "risk based surveillance"  OR  "risk based surveillances" ) ) | 30,482 |
| 1 | ( KEY ( «drinking  AND water»  OR  «water  AND supply» )  OR  TITLE-ABS ( "water*" ) ) | 3,287,810 |

Database: Web of Science Core Collection

Date: 10.04.2019

Number of identified articles: 740

Comment: Database covers the period 1987-2019 (=all years)

**# 4740#3 AND #2 AND #1**

Indexes=SCI-EXPANDED, SSCI, A&HCI, ESCI Timespan=All years

# 31,497,422TS=( "Waterborne Diseases" OR "Waterborne Disease" OR "Water borne Diseases" OR "Water borne Disease" OR outbreak* OR outbreaks OR waterborne OR "water borne" OR infection OR infections OR infectious* OR "water related disease" OR "water related diseases")

Indexes=SCI-EXPANDED, SSCI, A&HCI, ESCI Timespan=All years

# 257,077TS= («sentinel surveillance» OR «population surveillance» OR "syndromic surveillance" OR "syndromic surveillances" OR "Sentinel surveillance" OR "Sentinel surveillances" OR «early NEAR/1 outbreak» OR «early NEAR/1 outbreaks» OR "early warning" OR "early warnings" OR "risk based surveillance" OR "risk based surveillances" )

Indexes=SCI-EXPANDED, SSCI, A&HCI, ESCI Timespan=All years

# 12,273,034TS=( «drinking water» OR «water supply» OR water$)

Indexes=SCI-EXPANDED, SSCI, A&HCI, ESCI Timespan=All years

## Characteristics of included articles (n=18)

|  | Study ID | Country | Study design | Primary aim of study | Study period | Outbreak cause (aetiology) | Affected population | Syndrome/data for surveillance/detection for the outbreaks |
| --- | --- | --- | --- | --- | --- | --- | --- | --- |
| 1 | Andersson et al., 2013 | Sweden | Descriptive/analytical | Evaluate the efficiency of alternate data sources for early detection when comparing against nine outbreak, of which three large waterborne outbreaks | 2007-2011 | Gastrointestinal symptoms, food and waterborne outbreaks | Swedish population | Telephone triage  Web-queries  OCT pharmacy sales |
| 2 | Balter et al., 2005 | USA | Descriptive/analytical | Evaluation health’s departments use of syndromic surveillance for detection of waterborne outbreaks | November 2001-August 2004 | Gastrointestinal illness | New York City | Emergency department syndromic surveillance system |
| 3 | Bjelkmar et al., 2017 | Sweden | Descriptive/analytical | Summarize the outbreak investigations in Skellefteå and discuss potential for earlier detection using calls to a health advice line | Spring 2011 | *Cryptosporidium* | Skellefteå municipality | Phone calls to a health advice line |
| 4 | Burkom et al., 2011 | United States | Simulation | Test Bayesian Networkssoftware for early waterborne outbreaks including water quality data with health indicator data | NI | Waterborne diseases | American population | Fusion of water quality data (faecal indicator bacteria, chlorine, pH, conductivity and turbidity) with health monitoring data (“ESSENCE”) |
| 5 | Colón-Gonzales et al., 2018 | United Kingdom | Simulation | Investigate how the characteristic of different outbreaks affected outbreak detection and utility of syndromic surveillance to detect them | NI | Big pandemic influenza outbreak and localized outbreak of Cryptosporidiosis | England | Telehealth,  in-hours GP and out-of-hours,  ED visits |
| 6 | Coly et al., 2017 | France | Descriptive/analytical | Develop an integrated approach to detect any study clusters of acute gastrointestinal infection in geographical area with homogeneous exposure to drinking water | 2009-2012 | Acute gastrointestinal infection due to water exposure | Population in municipalities in a French region grouped by ‘geographical water supply network’ | Reimbursement of expenses for health care and drugs, geographical and population data, and environmental data based on common water exposure and water network |
| 7 | Cooper et al., 2006 | United Kingdom | Simulation | Modell whether calls about diarrhea (a proxy for cryptosporidiosis) exceeded a statistical threshold, thus alerting the surveillance team to the outbreak | Outbreak February – April 1997 | *Cryptosporidium* | London | Data from a health helpline (NHS Direct) |
| 8 | Edge et al, 2004 | Canada | Descriptive/analytical | Reviewing retrospective data from two Canadian waterborne outbreaks with different disease onset patterns | Spring 2001 | *Cryptosporidium* | Battlefords area of Saskatchewan and Walkerton, Ontario | Pharmacy OTC sales  ER visits |
| 9 | Heffernan et al., 2004 | United States | Descriptive/analytical | To provide a description of the syndromic surveillance in NYC since 1995 and their retrospective assessment | Since 1995 | Outbreaks of waterborne illness | New York City | Syndromic surveillance |
| 10 | Kirian et al., 2011 | United States | Descriptive/analytical | Evaluation of the availability of drugs sale to predict endemic and epidemic gastrointestinal disease in San Francisco area | Jan 2004 – Jul 2005 | Gastrointestinal illness | Population in the San Francisco Bay Area | Diarrheal remedy sales data  Diarrheal case counts from Health department |
| 11 | Mouly et al., 2016 | France | Descriptive/analytical | Comparative study of waterborne outbreaks from cohort studies with health administrative databases | Waterborne outbreak in 2010 and 2012 | *Campylobacter jejuni*, norovirus | Two cities in France | Health administrative databases, reimbursement of expenditures for medication (SNIIRAM) |
| 12 | Mouly et al., 2018 | France | Simulation | Evaluate the performance of an algorithm for waterborne outbreak detection through a simulation-based study | 2010-2013 | Waterborne illness | France | Daily baseline counts of acute gastrointestinal infections |
| 13 | Proctor et al., 1998 | United States | Descriptive/analytical | To examine surveillance data from eight sources which were available during the time of the outbreak | March-April 1993 | *Cryptosporidium* | Milwaukee, Wisconsin | Surveillance data from eight sources including water treatment plant, clinical laboratory diagnosis, nursing home diarrhoeal rates, ER, digit dialling telephone surveys, water utility complaint, school absentee and OTC sales). |
| 14 | Rambaud et al., 2016 | France | Descriptive/analytical | To assess the utility of using a health insurance database for the automated detection of waterborne outbreaks of acute gastroenteritis (AGE) | 2009–2012 | Unknown | Three French districts | Health administrative data (SNIIRAM) |
| 15 | Smith et al., 2010 | United Kingdom | Descriptive/analytical | Evaluate the value of syndromic surveillance in monitor small waterborne outbreak | June-July 2008 | *Cryptosporidium* | Northamptonshire | Data from syndromic surveillance system from direct telephone helpline and QSurveillance national surveillance system using clinical diagnosis data extracted from GP clinical information system |
| 16 | Xing et al., 2011 | United States | Simulation | To compare an enhanced C2 method with five regression models. to recognize increases in visit counts that might indicate an outbreak in automated surveillance system | May 2008 to April 2009 | Gastrointestinal illness | 16 cities in the US | Emergency department chief complaint data |
| 17 | Zhou et al., 2015 | United States | Simulation | Simulated alerting signals using syndromic data from 62 VHA hospitals for detection of outbreak | January 2010 through May 2011 | Gastrointestinal illness (according to diagnosis codes) | Population of US (Department of Veterans Affairs’ Veterans Health Administration (VHA)) | Outpatient VHA daily syndrome counts |
| 18 | Ziemann et al., 2013 | Europe | Descriptive/analytical | To propose a framework definition for specific syndromes and a SyS system design based on an inventory of sub-national emergency data availability in 12 countries | 2006-2009 (multiple countries) | Gastrointestinal illness (from diagnosis codes) | Population in Europe (several countries) | Routine emergency data |

## Assessment of Risk of Bias according to ROBINS-I

| **Agent Citation** | **Bias due to confounding** | **Bias in selection of participants** | **Bias in classification of interventions** | **Bias due to deviations from intended interventions** | **Bias due to missing outcome data** | **Bias in outcomes measurement** | **Bias in selection of results reported** | **Overall risk of bias** |
| --- | --- | --- | --- | --- | --- | --- | --- | --- |
| Andersson et al., 2013 | Low/moderate | Moderate/ serious | Low/moderate | N/A | N/A | N/A | serious | Moderate |
| Balter et al., 2005 | Serious | Low | Low | N/A | N/A | N/A | Moderate | Moderate/low |
| Bjelkmar et al., 2017 | Low/moderate | Moderate/serious | Moderate | N/A | N/A | N/A | serious | Moderate |
| Burkom et al., 2011 | Moderate | NI | Moderate/serious | N/A | N/A | N/A | serious | Serious |
| Colón-Gonzales et al., 2018 | Low | NI | Low/moderate | N/A | N/A | N/A | Moderate | Moderate/serious |
| Coly et al., 2017 | Moderate/serious | NI | Serious | N/A | N/A | N/A | Serious | Moderate/serious |
| Cooper et al., 2006 | Low/moderate | Moderate | Moderate | N/A | N/A | N/A | Serious | Moderate |
| Edge et al., 2006 | Serious | Low | Moderate | N/A | N/A | N/A | serious | Moderate/serious |
| Heffernan et al., 2004 | Serious | Low | Low | N/A | N/A | N/A | Moderate | Moderate/low |
| Kirian et al., 2011 | Moderate/low | Moderate | Moderate | N/A | N/A | N/A | Moderate | Moderate |
| Mouly et al., 2016 | Moderate/low | Moderate | Moderate | N/A | N/A | N/A | Serious | Moderate |
| Mouly et al., 2018 | NI | NI | Moderate | N/A | N/A | N/A | Serious | Moderate/serious |
| Proctor et al, 1998 | Moderate/low | Moderate | Moderate | N/A | N/A | N/A | Serious | Moderate |
| Rombaud et al, 2016 | Serious | Low | Moderate/serious | N/A | N/A | N/A | Serious | Moderate |
| Smith et al., 2010 | Moderate | Low | Moderate/serious | N/A | N/A | N/A | Moderate | Moderate |
| Zhou et al., 2015 | NI | NI | Moderate | N/A | N/A | N/A | Serious | Moderate/serious |
| Xing et al., 2011 | NI | NI | Moderate | N/A | N/A | N/A | Serious | Moderate/serious |
| Ziemann et al., 2014 | Serious | Low | Moderate | N/A | N/A | N/A | Serious | Moderate/serious |

## Table excluded articles with reasons

| Reason | Articles From literature search: | Screening of reference lists | Google Scholar search | Number |
| --- | --- | --- | --- | --- |
| Wrong study design | Beaudeau et al., 2008  Naumova et al., 2008  Paladini, 2004  Risebro et al, 2007 | Haas et al., 2011  Lombardo et al., 2003  Neill 2009 |  | n=7 |
| Wrong outcome (association/relationship) | Drayna et al., 2010  Elliott et al., 2016 | Beaudeau 1999  Beaudeau 2012  Bounoure et al., 2011  Curreiro et al., 2001  Derby et al., 2004  Edge et al, 2006  Egorov et al., 2003  Frosst et al., 2006  Gilbert et al., 2006  Hsieh et al., 2015  Hunter et al., 2005  Jackson et al., 2007  Morris et al., 1996  Noufaily et al. 2012  Tinker et al., 2010  Todkill et al., 2016 | Malm et al., 2013  Shortridge et al., 2014 | n=20 |
| Wrong study object |  | Kulldorff 2005 |  | n=1 |
| Wrong study context | El-Khatib et al., 2019  Nelesone et al., 2006  Pirard et al., 2015  Rosewell et al., 2013  Worwor et al., 2016 |  |  | n=5 |
| Duplication | Kirian et al., 2010 |  |  | n= 1 |
| Total |  |  |  | n=34 |
